# Reversible regulation of Cas12a activities by AcrVA5-mediated acetylation and CobB-mediated deacetylation

**DOI:** 10.1038/s41421-022-00396-0

**Published:** 2022-05-17

**Authors:** Xiaoman Kang, Lei Yin, Songkuan Zhuang, Tianshuai Hu, Zhile Wu, Guoping Zhao, Yijian Chen, Yong Xu, Jin Wang

**Affiliations:** 1 CAS Center for Excellence in Molecular Plant Sciences, Chinese Academy of Sciences, Shanghai, China; 2 University of Chinese Academy of Sciences, Beijing, China; 3 Tolo Biotechnology Company Limited, Shanghai, China; 4 Department of Clinical Laboratory, Shenzhen Second People’s Hospital & Institute of Translational Medicine/the First Affiliated Hospital of Shenzhen University Health Science Center, Shenzhen, China; 5 College of Life Sciences, Shanghai Normal University, Shanghai, China; 6 Institute of Antibiotics, Huashan Hospital, Fudan University, Shanghai, China; 7 Key Laboratory of Clinical Pharmacology of Antibiotics, National Health Commission, Shanghai, China; 8 Guangdong Key Laboratory of Systems Biology and Synthetic Biology for Urogenital Tumors, Shenzhen Second People’s Hospital/the First Affiliated Hospital of Shenzhen University, Shenzhen, China

**Keywords:** Molecular biology, Developmental biology

Dear Editor,

The clustered regularly interspaced short palindromic repeat (CRISPR)/CRISPR associated (Cas) system protects bacteria and archaea from mobile genetic elements (MGEs) such as bacteriophages^1^. The Cas effector can be guided by a CRISPR RNA (crRNA) to target the invading nucleic acids by base-pairing and then cleaves the target to provide the host with immunity^2,3^. To cope with the immunity pressure imposed by the host CRISPR system, phages have evolved different kinds of anti-CRISPR (Acr) systems to inactivate the Cas nucleases and close the host immunity system in turn^4^. The Acr proteins can directly interact with the Cas proteins to prevent the crRNA loading or target binding; alternatively, they possess enzymatic activities to cleave the crRNAs or post-translationally modify the Cas proteins^4^. Among them, there exists a recently reported GNAT-family acetyltransferase, namely AcrVA5, which is encoded by the MGEs and specifically inhibits the type V-A Cas12a effector through acetyl modification of a key lysine site. The AcrVA5-inactivated Cas12a loses the ability to interact with the protospacer adjacent motif (PAM) site and fails in protecting the hosts by cleaving the invading MGEs^5,6^. Through inactivating the Cas12a system, MGEs containing the *acrVA5* gene can effectively shut down the bacterial immunity system and have the potential to widely spread among the Cas12a-harboring hosts. However, to our great surprise, the *acrVA5* gene exists only in three *Moraxella bovoculi* strains which have lost their deacetylases (Supplementary Table S1). Therefore, it is reasonable to suspect that there may exist a competitive relationship between the acetyltransferase AcrVA5 and the widely distributed bacterial deacetylases^7^, which may reactivate Cas12a by deacetylation to protect the hosts from the invasion of MGEs harboring the *acrVA5* cassette.

We first performed the AcrVA5-mediated in vitro acetylation experiment and showed that the AcrVA5-acetylated *Lachnospiraceae bacterium* (Lb) Cas12a lost both *cis*- and *trans*-cleavage activities towards double-stranded target DNA (dsDNA) (Supplementary Fig. S1a, b), which was consistent with the previous findings^5,6^, however, the AcrVA5-treatment showed no effect on LbCas12a *trans*-cleavage activities when triggered by target single-stranded DNA (ssDNA) (Supplementary Fig. S1c). As the PAM site is only necessary for Cas12a to recognize target dsDNA but not ssDNA^8^, the above results further confirmed that AcrVA5-mediated acetylation prevented Cas12a from interacting with the PAM sequences in target dsDNA^5^.

Besides LbCas12a, we also analyzed Cas12a orthologs from *Francisella tularensis* subsp. novicida (FnCas12a) and *Acidaminococcus sp*. (AsCas12a) and demonstrated that AcrVA5 was able to inactivate both orthologs (Supplementary Figs. S2 and S3). Noticeably, AcrVA5 was ever shown to be ineffective against AsCas12a in a previous study^6^, however, we found that AsCas12a contained the conserved lysine residue (Supplementary Fig. S3c) and showed that the AcrVA5-mediated treatment led to the loss of both *cis-* and *trans-*cleavage activities of AsCas12a with target dsDNA.

Post-translational lysine acetylation plays an important role in diverse cellular processes in organisms from bacteria to human^9^. In bacteria, the NAD^+^-dependent sirtuin-type CobB deacetylates a large number of proteins and regulates the global acetylation level^7^. To test whether CobB can deacetylate the AcrVA5-treated Cas12a and reactivate its *cis*- and *trans*-cleavage activities, we then purified recombinant *E. coli* CobB and LbCas12a and performed the in vitro deacetylation assay. Based on the western blot results, Cas12a was successfully acetylated by AcrVA5 at the presence of acetyl-CoA, and the acetyl modification could be efficiently removed after being treated by CobB. Consistent with previous findings^7^, the CobB-mediated deacetylation stringently requires NAD^+^ as the cofactor (Supplementary Fig. S4). Accordingly, the AcrVA5-acetylated LbCas12a lost both *cis*- and *trans*-cleavage activities with target dsDNA but recovered both activities to a large extent after being deacetylated by CobB (Fig. 1a, b). In addition, we tested several Cas12a orthologs such as FnCas12a and AsCas12a and found CobB was able to deacetylate and reactivate both Cas12a orthologs (Supplementary Figs. S5–S8). It was worthy to mention that the successful reactivation of acetylated AsCas12a by CobB treatment once again proved AsCas12a as a target of AcrVA5 (Supplementary Fig. S3), while the reason for the distinct results between this work and the previous study^6^ was still unknown and could be an interesting question subject to further investigation. Based on the above results, one may conclude that AcrVA5 and CobB reversibly regulate both the *cis-* and *trans-*cleavage activities of Cas12a by modulating its acetyl status in vitro.

**Fig. 1:**
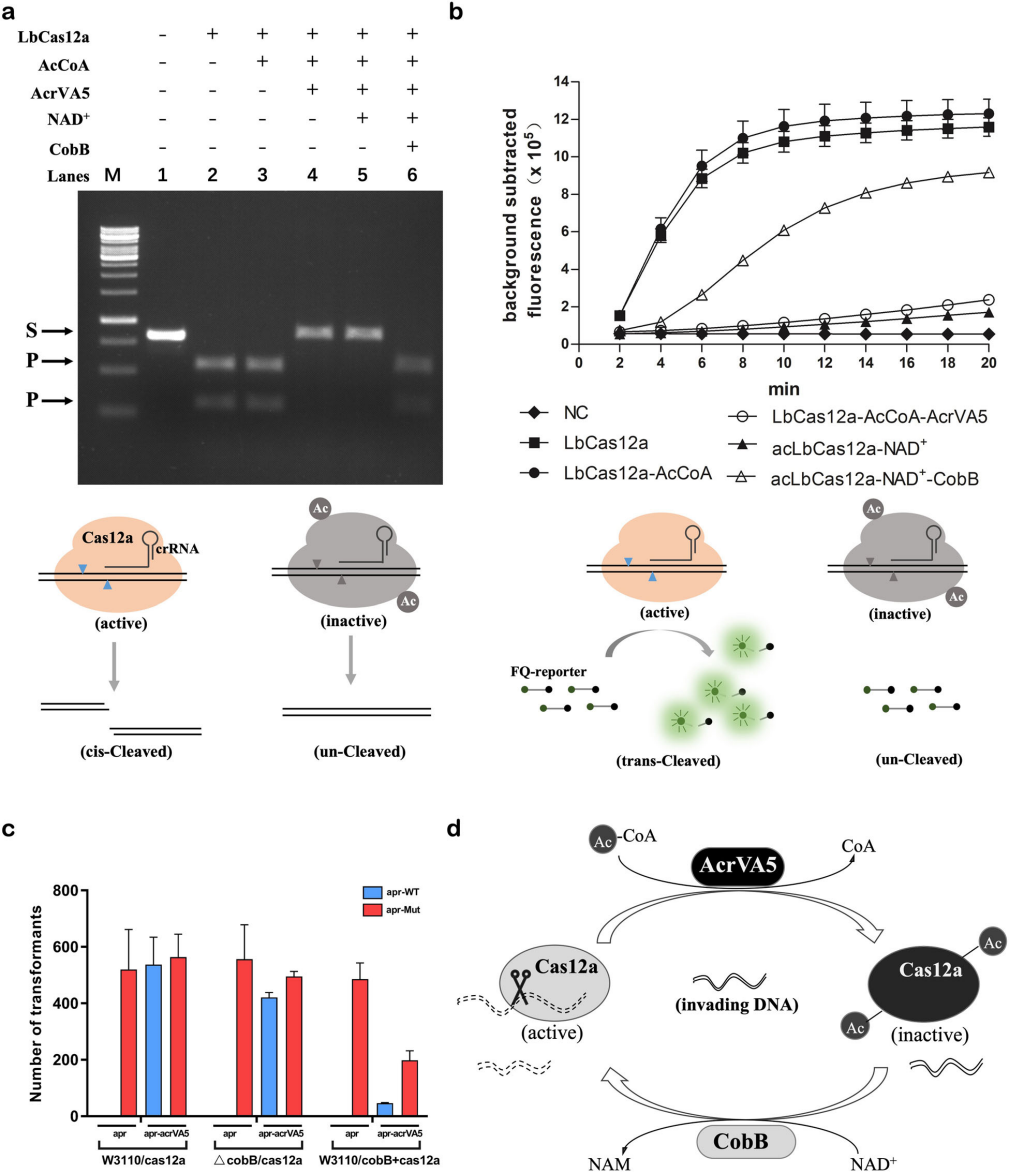
AcrVA5- and CobB-mediated reversible regulation of Cas12a activities through acetylation and deacetylation. **a** The LbCas12a *cis*-cleavage experiment with target dsDNA. As illustrated below, active Cas12a successfully cleaved target dsDNA into two pieces by with the guidance of crRNA, however, acetylated Cas12a became inactivated and lost the *cis*-cleavage activities against target dsDNA. M, 1-kb DNA ladder (Thermo Fisher Scientific); S, dsDNA substrate; P, Cas12a *cis*-cleaved products. **b** The LbCas12a *trans*-cleavage experiment with target dsDNA. As illustrated below, the *trans*-cleavage activities of active Cas12a proteins were triggered by target dsDNA, *trans*-cleaving the fluorescence-quencher reporter (FQ-reporter), and illuminating fluorescent signals. However, once Cas12a was acetylated, it became inactivated and lost the *trans*-cleavage activities in the presence of target dsDNA. Fluorescence signal was collected with a real-time qPCR machine and values were shown with background signal subtracted. NC, the negative control reaction with no target added; LbCas12a, reaction using untreated LbCas12a; LbCas12a-AcCoA, reaction using LbCas12a treated with acetyl-CoA only; LbCas12a-AcCoA-AcrVA5, reaction using LbCas12a treated with AcrVA5 at the presence of acetyl-CoA; acLbCas12a, AcrVA5-acetylated LbCas12a; acLbCas12a-NAD^+^, acetylated LbCas12a treated with NAD^+^ only; acLbCas12a-NAD^+^-CobB, acetylated LbCas12a treated with CobB at the presence of NAD^+^. **c** Analysis of the physiological role of CobB in protecting the host from invading foreign *apr* reporter plasmid. W3110/cas12a, the wild type expressing the LbCas12a/crRNA complex; ΔcobB, the *cobB* null mutant expressing the LbCas12a/crRNA complex; W3110/cobB+cas12a, the wild type expressing the LbCas12a/crRNA complex as well as the CobB. apr-WT, reporter plasmid with the wild type *apr* gene; apr-Mut, reporter plasmid with the mutant *apr* gene that can escape from the cleavage by Cas12a. **d** A regulatory model of AcrVA5- and CobB-mediated reversible regulation of Cas12a activities. The acetyltransferase AcrVA5 uses acetyl-CoA to acetylate Cas12a, inactivating Cas12a as well as the host resistance system against MGEs, however, the host possesses the NAD^+^-dependent CobB, which deacetylates and reactivates Cas12a and provides the host a secondary safeguard to the invading nucleic acids.

We further explored the physiological role of CobB in protecting the Cas12a-containing host from the invasion of MGEs that harbor the *acrVA5* gene. We first constructed a CRISPR plasmid that expressed both LbCas12a and a crRNA targeting a specific sequence in the apramycin resistance gene (*apr*) and transformed it into *E. coli* strains with or without a high level of CobB expression (Supplementary Fig. S9). Because of the low expression level of the native *cobB* gene in *E. coli*^10^ (Supplementary Fig. S10), the wild type W3110 as well as the *cobB*-deleted strains were considered as the *cobB*-negative host, while the *cobB* overexpression strain was the *cobB*-positive host. To prepare a reporter plasmid without the targeting sequence of the Cas12a/crRNA complex, we mutated the Cas12a-targeting DNA sequence in the *apr* gene without changing the amino acid sequence as well as the apramycin resistance (Supplementary Fig. S11), obtaining a mutated *apr* gene. Because the nonsense mutation changed both the PAM site and the guide sequence, Cas12a failed to cleave the target sequence both in vitro (Supplementary Fig. S11b). As a result, the reporter plasmid with mutant *apr* effectively escaped from the host defense system and showed high transformation efficiencies in an *acrVA5*-independent manner (Fig. 1c), which was therefore employed as a control of the transformation assay.

The reporter plasmid containing the wild type *apr* gene was then transformed into *E. coli* strains expressing Cas12a to mimic the invasion of MGEs, and as expected, in the absence of *acrVA5* gene, no transformants could be obtained in all tested strains independent on the expressional level of CobB, representing the invasion of the foreign plasmid could be completely blocked by the hosts (Fig. 1c). However, if the reporter plasmid contained both *apr* and *acrVA5*, mimicking MGEs harboring the *acrVA5* cassette, the high transformation efficiency of the reporter plasmid was obtained in the *cobB*-negative hosts, which was consistent with the above findings that AcrVA5 effectively inactivated Cas12a. As the overexpression of CobB in the *cobB*-positive host could deacetylate and reactivate the AcrVA5-acetylated Cas12a, the transformation of the reporter plasmid was largely blocked. As expected, the efficiency of the reporter plasmid was still much lower than the control, although overexpression of CobB and AcrVA5 may severely reduce the *E. coli* transformation efficiencies (Fig. 1c). Based on the above results, one may conclude that AcrVA5 and CobB reversibly regulated the Cas12a activities in vivo through acetylation and deacetylation, respectively.

As the AcrVA5-mediated acetyl modification of Cas12a has been considered as an effective anti-Cas12a strategy by MGEs^5,6^, the CobB-mediated deacetylation reactivation of Cas12a through modification can be taken as both a prevention strategy to the anti-CRISPR elements and a safeguard to the CRISPR defense system, protecting hosts from the invading of the *acrVA5*-containing MGEs (Fig. 1d). Moreover, this study highlights the potential to create more complexed systems to regulate the Cas12a cleavage activities, which may facilitate both in vivo gene editing and in vitro target nucleic acid detection^11^.

Besides Cas12a, AcrVA5 may function as a broad-spectrum acetyltransferase and influences cellular metabolic processes. We next performed the in vitro western blot analysis through using *E. coli* cellular extracts and purified AcrVA5 and found a large number of proteins could be acetylated by AcrVA5 (Supplementary Fig. S12). Similarly, after overexpression of AcrVA5 in *E. coli*, the whole cellular acetylation level was greatly increased, and the acetylated proteins could then be efficiently deacetylated by CobB at the presence of NAD^+^ (Supplementary Fig. S13). With the employment of mass spectrometry, we further characterized 2688 sites and 1101 proteins as potential AcrVA5 targets (Supplementary Table S2). Probably due to the small protein size and the reduced steric hindrance in turn, AcrVA5 showed no obvious target sequence preference but favored positively charged amino acids such as lysine, arginine, and histidine (Supplementary Fig. S14). Considering the extensive targets of AcrVA5, one may imagine that CobB and AcrVA5 compete for control of both the host immune system and beyond. Moreover, the Dsr proteins, which contain the deacetylase domains, were recently found to protect hosts against the invading of dsDNA phages^12^, and the present study may possibly provide a clue for the unclear mechanisms.

Taken together, one could infer that the wars between invading MGEs and the microbial hosts will never end, and besides acetyl- and deacetyl-modification, there probably exist other kinds of competitive mechanisms subject to future investigation. While just as the Chinese idiom goes, one can believe a vice rises one foot, but virtues rise ten.

## Supplementary information

Supplementary Information
